# Analysis of *FRAME* data (A‐FRAME): An analytic approach to assess the impact of adaptations on health services interventions and evaluations

**DOI:** 10.1002/lrh2.10364

**Published:** 2023-03-15

**Authors:** Heather Z. Mui, Cati G. Brown‐Johnson, Erika A. Saliba‐Gustafsson, Anna Sophia Lessios, Mae Verano, Rachel Siden, Laura M. Holdsworth

**Affiliations:** ^1^ Division of Primary Care and Population Health, Department of Medicine School of Medicine, Stanford University Palo Alto California USA

**Keywords:** adaptation, COVID‐19 pandemic, health services research, implementation science, quality improvement

## Abstract

**Introduction:**

Tracking adaptations during implementation can help assess and interpret outcomes. The framework for reporting adaptations and modifications‐expanded (FRAME) provides a structured approach to characterize adaptations. We applied the FRAME across multiple health services projects, and developed an analytic approach to assess the impact of adaptations.

**Methods:**

Mixed methods analysis of research diaries from seven quality improvement (QI) and research projects during the early stages of the COVID‐19 pandemic. Using the FRAME as a codebook, discrete adaptations were described and categorized. We then conducted a three‐step analysis plan: (1) calculated the frequency of adaptations by FRAME categories across projects; (2) qualitatively assessed the impact of adaptations on project goals; and (3) qualitatively assessed relationships between adaptations within projects to thematically consolidate adaptations to generate more explanatory value on how adaptations influenced intervention progress and outcomes.

**Results:**

Between March and July 2020, 42 adaptations were identified across seven health services projects. The majority of adaptations related to training or evaluation (52.4%) with the goal of maintaining the feasibility (66.7%) of executing projects during the pandemic. Five FRAME constructs offered the most explanatory benefit to assess the impact of adaptations on program and evaluation goals, providing the basis for creating an analytic approach dubbed the “A‐FRAME,” analysis of FRAME data. Using the A‐FRAME, the 42 adaptations were consolidated into 17 succinct adaptations. Two QI projects discontinued altogether. Intervention adaptations related to staffing, training, or delivery, while evaluation adaptations included design, recruitment, and data collection adjustments.

**Conclusions:**

By sifting qualitative data about adaptations into the A‐FRAME, implementers and researchers can succinctly describe how adaptations affect interventions and their evaluations. The simple and concise presentation of information using the A‐FRAME matrix can help implementers and evaluators account for the influence of adaptations on program outcomes.

## 1 INTRODUCTION

The implementation of health service interventions into real‐world settings is dynamic and messy; rarely are evidence‐based interventions implemented into new settings without the need for tailoring to local conditions.^1^, ^2^, ^3^ Although changes to interventions and projects commonly occur, systematic documentation of adaptations made during implementation to fit the local context is lacking.^4^, ^5^, ^6^ Not accounting for the influence of adaptations could potentially lead to misinterpreting evaluation findings and drawing erroneous conclusions about program effectiveness.^7^ Understanding how adaptations affect interventions and their goals is essential for continuously improving systems to ensure that programs are meeting their desired outcomes in the expected way.

One tool for systematically categorizing adaptations made to interventions is the Framework for Reporting Adaptations and modifications‐expanded (FRAME).^4^ The FRAME describes 10 constructs that capture the process of creating adaptations (eg, *when* the adaptation occurred, *who* was involved in the decision), the types of adaptations (eg, *what is modified*), and why adaptations were made (eg, *goals*, *reasons*). Researchers and implementers have used the FRAME to classify intervention adaptations to help assess intervention effectiveness in new settings^8^ and to document and describe implementation strategies.^9^, ^10^ While systematic description of adaptations using the FRAME is helpful, there is greater potential for harnessing the data to enhance the explanatory value of studies. The purpose of tracking adaptations is to not only identify changes, but to understand what changes influenced intervention outcomes and how. However, there is no guidance for how researchers can analyze data about adaptations to meaningfully contribute to interpreting data from implementation and effectiveness studies.

While it is anticipated that all interventions require adaptations when implementing in new settings,^1^ the COVID‐19 pandemic was a distinctive experience for healthcare systems affecting all aspects of delivery systems, leading to an enormous amount of rapid innovation and adaptation.^11^, ^12^, ^13^, ^14^, ^15^ The unique context of the pandemic provided an opportunity to examine adaptations across multiple health services research and quality improvement (QI) projects using the FRAME and develop a process for analyzing the documented adaptations.

### 1.1 Aims and research questions

The aim of this study was to apply the FRAME to characterize adaptations made to health services projects within one academic research center during the early stages of the COVID‐19 pandemic and develop a process to analyze data about adaptations. Our specific research questions were:

1. How were interventions and their evaluations modified in response to the COVID‐19 pandemic?
2. How can information captured in the FRAME be utilized to help evaluators make sense of the impact of adaptations on intervention and evaluation outcomes?

Following our analysis, we introduce a novel approach for analyzing the impact of adaptations captured using the FRAME.

## 2 METHODS

We conducted a mixed methods analysis of the project adaptations captured in research diaries during the early stages of the COVID‐19 pandemic.

### 2.1 Sample

We collected research diaries from active QI and research projects within one health services research unit in an academic medical school. As our aim was to identify adaptations made due to the COVID‐19 pandemic, we only included projects which were either: preparing for implementation, preparing to collect data, or collecting data. We excluded projects in the analysis or reporting phases. Seven projects were considered active and included in our analysis.

### 2.2 Data collection

We created research diaries for each project to record daily and/or weekly observations of project events in Microsoft Excel. Entries included the date of the event, summary of the event, whether the event related to project implementation or evaluation, how the researcher was made aware of the event (eg, email, meeting, observation, or interview) and a link to the information for reference. The research diaries were created specifically to align tracking practices across projects. These research diaries formed the basis for abstracting data related specifically to adaptations.

Project teams nominated one researcher, all of whom are authors on this paper, from each project to identify adaptations within project diaries and extract adaptations into an Excel matrix for further analysis (referred to as “FRAME project matrices”). The nominated researcher was part of the QI or research project team and thus considered knowledgeable in deciding whether a project change was an adaptation or not. To guide the identification of adaptations, we used Wiltsey‐Stirman and colleagues' definition of modifications and adaptations: “Modifications can include adaptations, which are planned or purposeful changes to the design or delivery of an intervention, but they can also include unintentional deviations from the interventions as originally designed” (Wiltsey‐Stirman et al, 2013, p. 2)^5^. For simplicity, we refer to all changes as “adaptations.” Researchers also referred to the FRAME codebook (available online: https://med.stanford.edu/fastlab/research/adaptation.html) to guide decision making, as this provided further information on the different elements of adaptations that might be considered. Where researchers were not sure whether to classify an event as an adaptation, they discussed this with the project implementers, when possible, to arrive at a final decision.

**FIGURE 1:**
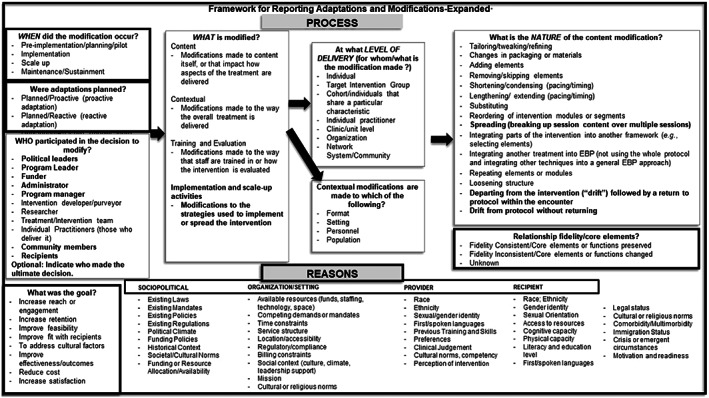
The framework for reporting adaptations and modifications‐expanded (FRAME), from Wiltsey‐Stirman et al 2019^4^

Using Wiltsey‐Stirman and colleagues' 2019^4^ and 2013^5^ articles, the FRAME figure outline (Figure 1), and the FRAME coding manual, each nominated researcher categorized the adaptations for their respective project using the categories within each FRAME construct and provided a brief explanation for the categorization. When an adaptation did not fit into any of the existing FRAME categories, the researcher created a new category and provided a description. The researchers representing all seven projects met biweekly during the 4‐month period to discuss new project adaptations and align on use and understanding of the FRAME categories. We found some inconsistencies between the figure and manual; these discrepancies were resolved through group discussion. Each row in the FRAME project matrices represented one adaptation, and each FRAME construct was a column heading. Additional columns were added to facilitate cross‐project analysis: project name, adaptation name, and description of the original project component so researchers external to the project could interpret the adaptation. We also included a column to capture the researcher's assessment of the short‐term impact of the adaptation (Rabin et al^6^), and an open category for researchers to note information not represented in the FRAME (see Table S1 in supplemental material). FRAME project matrices were reviewed at two timepoints by HZM and LMH for reliability in researcher understanding and completeness. Any discrepancies were discussed with the project researcher to ensure similar descriptions were categorized in the same way, working together to clarify or recategorize the adaptation.

### 2.3 Data analysis

Our analysis process consisted of three steps. Step 1, we used Excel to calculate the frequency of adaptation categories by FRAME construct *across all projects* to see the range of FRAME categories represented by the identified adaptations. Step 2, we reviewed adaptations *within projects* individually to qualitatively assess the impact adaptations had on project goals. To assess impact, we examined the categories for each adaptation *within projects* in relation to the Rabin et al^6^ construct of perceived short‐term impact, including impacts to reach, adoption, and implementation, and the FRAME construct “relationship to fidelity”, whether the adaptation preserved or altered the intervention's core elements or functions. The researcher used their depth of knowledge about the intervention to determine whether the adaptation had any short‐term impact or impact on fidelity. We looked for patterns in researcher's descriptions to identify whether information captured in any construct provided more or less explanation as to whether an adaptation was rated as having an impact or not, for example, whether a change in the *content* vs a change in the *format* was driving the impact on the intervention as a whole. Step 3, we looked to see whether adaptations within projects were linked or related, and thus could be consolidated into one adaptation with more explanatory value for the project. For example, Project F was a national, multi‐site study where multiple sites reported shifting recruitment of patients from in‐person to telephone, and thus individually reported recruitment adaptations were consolidated into one adaptation. In assessing the cumulative impact of adaptations, we merged and refined the FRAME constructs into the conceptual domains of *what* was modified, *how* it was modified, *why* it was modified, and whether the core function was altered (ie, *impact*).

## 3 RESULTS

Table 1 describes the seven projects included in our analysis. Four projects evaluated local level QI initiatives within one hospital system; one was a research study which included multiple hospital systems within the San Francisco Bay Area, and two were research studies involving multiple health systems across the United States. All studies used mixed methods in their evaluation strategies.

**TABLE 1:** Project descriptions

Project	Level	Approach	Description and aim
A	Local, single institution	Quality improvement	Home‐based senior care program to increase access to routine primary care and social work management by homebound older adults in the home
B	Local, single institution	Quality improvement	Video visit roll‐out in ambulatory neurology clinics to assess acceptability, appropriateness, and sustainability of video visits
C	Local, single institution	Quality improvement	Platform and process to reduce operating room case cycle time
D	Local, single institution	Quality improvement	Hospital‐based predictive model to identify patients appropriate for advanced care planning
E	Regional, multi‐institution	Research	Implementation of palliative care programs to increase reach, reduce suffering, and increase goal concordant care
F	National, multi‐institution	Research	Perioperative palliative care to support upper gastrointestinal cancer patients
G	National, multi‐institution	Research	Collaborative to increase supportive care practices for end‐stage kidney disease in dialysis centers

Entries into the research diaries from March 11, 2020 to July 1, 2020 were included in the FRAME project matrices for analysis as this was perceived to be approximately the first wave of the pandemic in the United States. A total of 42 adaptations across the seven projects were identified. Three projects (B, F, G) recorded nine adaptations, one project had seven (A), one had six (E), and two projects (C, D) had one adaptation each. FRAME categories are indicated by italics throughout the remainder of the paper.

### 3.1 Across project analysis: frequency of adaptations by category (step 1)

Table 2 presents the frequencies of adaptations by FRAME categories by project and the total across projects. Here, we describe the categories of highest frequency per FRAME construct. Most adaptations occurred during *implementation* (33, 78.6%) and were *unplanned/reactive* (28, 66.7%). Decisions to make adaptations often involved *researcher*s (20, 47.6%), *administrators* (17, 40.5%), and/or *intervention developers* (13, 31.0%), while those whom the adaptations affected were primarily at the *clinic or unit level* (13, 31.0%) or *target intervention group* (11, 26.2%). Changes to *training or evaluation* plans (22, 52.4%) were the most frequently reported type of adaptation, with evaluation making up all but one of those adaptations. Evaluation adaptations included pauses in recruitment and data collection, adapting methods for remote forms of data collection (eg, conducting interviews via video or phone instead of in‐person), adding interview questions to evaluations to explore the impact and perceptions of the pandemic, and changing data collection methods (eg, interviews in lieu of observations). Most adaptations were considered *fidelity consistent* (25, 59.5%)*; fidelity inconsistent* adaptations (15, 35.7%) were those that altered the ability to deliver the intervention as intended (eg, missed trainings) or altered the methodological strength of evaluations. The goal of most adaptations was to *improve feasibility* (28, 66.7%) of executing projects. Unsurprisingly, the *reason* most adaptations were made was due to the pandemic (20, 47.6%) and resulting mandates (8, 19.0%) (ie, shelter‐in‐place orders), although this was not previously represented in the FRAME; consequently, the categories of *pandemic or other crisis* and *emergent/new mandates* were added to the *sociopolitical* reasons. Step 1 indicated that some of the detailed categories were not used, but that those that were selected often appeared multiple times in a project and single adaptations often had multiple categories selected.

**TABLE 2:** Frequency of adaptations across seven projects (A‐G) coded to FRAME categories.

FRAME construct	Categories	A (n = 7)	B (n = 9)	C (n = 1)	D (n = 1)	E (n = 6)	F (n = 9)	G (n = 9)	Total (n = 42)
When did the modification occur?^a^	Implementation	7	4	‐	‐	4	9	9	33
	Pre‐implementation	‐	5	1	1	2	‐	‐	9
Were adaptations planned?	Unplanned/reactive	7	7	1	1	4	4	4	28
	Planned/proactive	‐	2	‐	‐	2	5	5	14
Who participated in the decision to modify?^a^ ^,^ ^b^	Researcher(s)	2	8	1	1	2	3	3	20
	Administrator(s)	1	6	1	1	2	6	‐	17
	Intervention developer/purveyor	‐	6	1	‐	‐	‐	6	13
	Treatment/intervention team	4	‐	‐	‐	1	‐	3	8
	Individual practitioner	‐	‐	‐	‐	‐	‐	4	4
	Program leader	3	‐	‐	‐	‐	‐	‐	3
	Program manager	‐	‐	1	‐	‐	‐	‐	1
	Unsure^c^	‐	‐	‐	‐	2	‐	‐	2
Level of delivery (for whom/what the modification is made)?^a^	Clinic or unit level	3	1	1	‐	2	2	4	13
	Target intervention group	3	4	‐	‐	‐	3	1	11
	Organization	‐	‐	‐	1	2	4	‐	7
	Network system or community	‐	‐	‐	‐	2	‐	4	6
	Cohort	‐	2	‐	‐	‐	‐	‐	2
	Individual practitioner	1	‐	‐	‐	‐	‐	‐	1
	N/A^c^	‐	2	‐	‐	‐	‐	‐	2
What is modified?	Training and evaluation	2	8	‐	‐	3	6	3	22
	Contextual^d^	3	‐	‐	‐	2	‐	3	8
	Project stop or pause^c^	1	‐	1	1	‐	3	‐	6
	Implementation and scale‐up activities	‐	1	‐	‐	1	‐	2	4
	Content^e^	1	‐	‐	‐	‐	‐	1	2
Relationship to fidelity?	Fidelity consistent	4	5	‐	‐	2	9	5	25
	Fidelity inconsistent	3	4	‐	‐	4	‐	4	15
	Unknown/not applicable	‐	‐	1	1	‐	‐	‐	2
What was the goal?^a^ ^,^ ^b^	Improve feasibility	2	7	1	1	3	9	5	28
	Increase reach or engagement	‐	5	1	‐	1	‐	‐	7
	Increase retention	2	‐	‐	‐	‐	‐	4	6
	Improve fit with recipients	2	‐	‐	‐	‐	‐	1	3
	Improve effectiveness/outcomes	1	‐	‐	‐	1	‐	1	3
	Reduce risk^c^	3	‐	‐	‐	‐	‐	‐	3
	Reduce cost	‐	1	‐	‐	1	‐	‐	2
Reasons^a^ ^,^ ^b^	*Sociopolitical*								
	Pandemic or other crisis^c^	4	‐	1	1	2	3	9	20
	Emergent/new mandate^c^	1	5	‐	‐	1	1	‐	8
	Funding policies	1	‐	‐	‐	‐	‐	‐	1
	Organization/setting								
	Competing demands or mandates	‐	2	1	1	1	6	‐	11
	Regulatory or compliance	‐	3	‐	‐	2	1	‐	6
	Social context	‐	2	‐	‐	2	‐	‐	4
	Location or accessibility	‐	1	‐	‐	‐	2	‐	3
	Available resources	1	2	‐	‐	‐	‐	‐	3
	Time constraints	‐	1	‐	‐	1	‐	‐	2
	*Provider*								
	Clinical judgement	1	‐	‐	‐	‐	‐	‐	1
	*Recipient*								
	Crisis or emergent circumstances	4	‐	‐	‐	‐	‐	‐	4
	Access to resources	1	‐	‐	‐	‐	‐	‐	1

^a^ Only FRAME constructs and categories coded for the projects are represented. The number of categories not included by construct are: “When did the adaptation occur” = 2, “Who participated in the decision to modify” = 4, “Level of delivery” = 1, “What was the goal” = 2, *“*Reasons” = 32 (sociopolitical = 8; organization/setting = 4; provider = 8; recipient = 12).

^b^ Constructs allowed multiple categories to be selected per adaptation; totals can be greater than the number of adaptations.

^c^ Indicates category added to the FRAME (Wiltsey‐Stirman et al., 2019).

^d^ Within the *contextual* category, 7 adaptations were to the *format* (3 each in projects A and G, 1 in project F), and 1 adaptation was to *personnel* (project F).

e Within the *content* category, 1 adaptation involved *adding elements* (project A), and 1 adaptation involved *loosening structure* (project G).

Six adaptations, which all related to projects stopping or pausing activity, did not fit into any of the FRAME's four “What is modified” category types; we therefore added a fifth category: *project stop or pause*. Projects C and D, both QI projects in the pre‐implementation phase, stopped altogether due to organizational decisions to focus efforts on pandemic relevant projects. Project C abandoned their plans to reduce operating room case cycle time and instead shifted to testing telehealth visits for surgical cases. Project D stopped the development of a tool to identify patients appropriate for advance care planning to focus on an algorithm to detect clinical deterioration.

### 3.2 *A*nalysis of *FRAME* data: assessing the impact of adaptations within projects (steps 2 and 3)

During step 2, we found that the FRAME constructs that provided the most explanatory details as to why an adaptation was rated as having an impact or not on the project were “What is modified”, “What is the nature of the content modification,” “What was the goal,” “Reasons,” and “Relationship to fidelity.” The remaining FRAME constructs did not offer any explanatory information for how project goals and core function were impacted. In addition, we found that an essential starting point for assessing the impact of the adaptation was first understanding the original plan, along with the perceived short‐term results of the adaptation. Step 3 combined adaptations within projects which appeared to cumulatively contribute to the same identified impact on a project. This process of synthesizing discrete adaptations into a more complete picture of the nature and impact of the change led us to refine the presentation of the FRAME constructs into what we call the analysis of FRAME data (A‐FRAME) matrix. Table 3 describes steps 2 and 3 in the development of the A‐FRAME matrix, including which FRAME constructs were not included, merged, or refined, and new concepts added. The A‐FRAME is the analytic approach used for analyzing the data captured by the FRAME to assess the impact adaptations have on a project's goals and function, and can also be used to succinctly summarize adaptations and their impacts for use by implementers and researchers (see Table 4).

**TABLE 3:** Development of the analysis of framework for reporting adaptations and modifications‐expanded data (A‐FRAME) matrix from the FRAME

FRAME construct	FRAME categories	FRAME constructs and categories used or modified to assess impact	New concepts added	A‐FRAME matrix domains
When did the modification occur?	Pre‐implementation/planning Implementation Scale‐up Maintenance/sustainment	Not included		N/A
Were adaptations planned?	Planned/ proactive Unplanned/ reactive	Not included		N/A
Who participated in the decision to modify?	11 categories (eg, administrator, intervention developer/purveyor)	Not included		N/A
What is modified?	Content Contextual Training and Evaluation Implementation and scale‐up activities	Reorganized into thematic groups: Intervention (content, contextual, training, implementation activities) Evaluation Project discontinued		What was modified?
At what level of delivery (for whom/what is the modification made)?	7 categories (eg, target intervention group, clinic/unit level)	Not included		How was it modified?
Contextual modifications are made to which of the following?	Format Setting Personnel Population	Used as described		
What is the nature of the content modification?	15 categories (eg, adding elements, lengthening/extending)	Expanded to also categorize adaptations in training and evaluation and implementation activities, with additional description.		
What was the goal?	8 categories (eg, increase reach or engagement, improve feasibility)	Added “reduce risk” option.		Why was it modified?
Reasons	Sociopolitical 9 options (eg, existing laws, societal/ cultural norms) Organization/Setting 10 options (eg, available resources, time constraints) Provider 8 options (eg, preferences, clinical judgement) Recipient 14 options (eg, access to resources, cognitive capacity)	Added two other options to sociopolitical: “Pandemic or other crisis” “Emergent/new mandate”		
Relationship fidelity/core elements?	Fidelity consistent/ core elements or functions preserved Fidelity inconsistent/ core elements or functions changed Unknown	Greater emphasis given to core function (purpose and goals) rather than core elements (components or activities).		Impact: Was the core function altered? How?
			What were the perceived short‐term results of the adaptation? (Rabin et al.^6^ )	
			Project description: describe intervention and evaluation plans and aims	Project summary

**TABLE 4:** Exemplar use of the analysis of framework for reporting adaptations and modifications‐expanded data (A‐FRAME) matrix for seven projects.

Project summary	What was modified? (thematic group)	How was it modified?	Why was it modified?	Impact: Was the core function altered? How?
Project A: Home‐based senior care program aimed at providing primary care to homebound patients at their place of residence. The program evaluation sought to examine patient and caregiver outcomes using surveys, observations of home visits, and patient and caregiver interviews	Intervention: Delivery	In‐person home visits shifted to phone outreach and video visits; paused enrollment of new patients	Limiting in person contact to reduce risk of virus exposure and spread	Yes, impacted access: limited access to care
	Intervention: Delivery	Changed visit protocol to include protective equipment; provided COVID‐19 testing at patient's home	Improve safety and access to testing to reduce risk of virus exposure and spread	No
	Evaluation: Data collection	Added questions about COVID‐19 to interviews; conducted phone interviews in lieu of in‐home observations with interviews	Wanted to understand impact of COVID‐19 on patients; limit in person contact to reduce risk	No
Project B: Neurology clinic initiative to conduct a phased gradual roll‐out of video visits for ambulatory care appointments to understand acceptability, appropriateness, and sustainability. Mixed methods data collection and analysis with patient and provider surveys, provider interviews, and observations	Intervention: Delivery	Shifted to 100% video visits rather than a phased gradual roll‐out	In‐person appointments reduced due to COVID‐19; virtual visits preferred operationally	No
	Evaluation: Study design	Stepped‐wedge design changed to 100% rapid roll‐out of video visits with no comparison group	(same as above)	Yes, impacted knowledge on effectiveness: design weakened
	Evaluation: Data collection	Conducted phone interviews in lieu of observations with interviews; patient surveys changed to interviews; data collection expanded to include more participant views; rapid data analysis rather than full coding	Unable to visit clinics in person; needed more in‐depth and rapid feedback to reach goals on time	No
Project C: Initiative to develop a platform and workflow to reduce operating room case cycle times (the total time of the procedure plus the time it takes to clean and set‐up the room for the next surgery)	Project discontinuation	Project suspended and resources reallocated	Regional mandates to cancel elective surgeries; project deprioritized in favor of a project related to changes due to COVID‐19 (telehealth)	Project not completed
Project D: Hospital‐based initiative to implement a palliative predictive model to identify patients for advance care planning (ACP) and a workflow to support providers in having ACP conversations	Project discontinuation	Project suspended and resources reallocated	Project deprioritized by operational leaders in favor of a project to address COVID‐19 (clinical deterioration)	Project not completed
Project E: Palliative care program expansions or pilots at seven health systems to provide comprehensive palliative care for people with serious illness with the goals of increasing reach, reducing suffering, and increasing goal concordant care. Program components included hiring, training staff, and educating patients and referring providers. Mixed methods evaluation with aggregate patient data, interviews with providers, and site visits to assess effectiveness and implementation outcomes	Intervention: Training	Delayed or cancelled trainings needed to upskill staff	Unable to meet in person and trainings not yet moved to virtual format	Yes, impacted implementation: reduced ability to deliver intervention
	Intervention: Staffing	Hiring freezes resulted in incomplete and understaffed teams	Financial uncertainty at the organizational level	Yes, impacted reach: limited capacity to deliver intervention
	Intervention: Delivery	In‐person visits shifted to video or telephone visits	Shelter in place orders and limiting in‐person contact	No
	Evaluation: Data collection	Postponed and changed data collection to virtual format; added interviews to ask about COVID‐19 impact	Unable to meet in person; wanted to understand impact of COVID‐19 on programs	No
Project F: Palliative care intervention during the perioperative period for patients with upper gastrointestinal cancer seeking curative treatment. Phase of research included patient caregiver recruitment, and data collection of surveys and interviews to understand patient experience	Intervention: Delivery	Because surgeries were cancelled, there were no eligible patients for the study and the intervention was paused until surgeries resumed	Regional mandates to cancel non‐essential surgeries to prepare for surge	No
	Evaluation: Recruitment and data collection	Recruitment paused as surgeries were suspended; once resumed, recruitment and data collection shifted to remote methods	Regional mandates to cancel non‐essential surgeries; unable to physically be in clinics	No
Project G: A learning collaborative to promote supportive care practices in dialysis centers for patients with end‐stage kidney disease. Evaluation of the collaborative and supportive care practices included collection of health utilization data, observation of webinars and learning sessions, site visits to dialysis centers, surveys, and interviews	Intervention: Training	Content changed to focus on the pandemic rather than planned content; in‐person learning session moved to virtual format	Focus shifted to COVID‐19; unable to travel	Yes, impacted participation/reach: content changed and lower virtual session attendance
	Intervention: Delivery	Some dialysis centers stopped identifying seriously ill patients and having goals of care discussions	Focus shifted to managing patient care and minimizing time in the dialysis center	Yes, impacted adoption: intervention activities not completed
	Evaluation: Data collection	Site visits cancelled; survey timing and content for patients changed to ask questions about COVID‐19	Unable to travel; wanted to understand impact of COVID‐19 on implementers	No

Sifting FRAME data from the project matrices into the A‐FRAME, we identified three thematic groups for the types of adaptations made to the seven projects: (a) project discontinuation, (b) intervention adaptation, and (c) evaluation adaptation. As shown in Table 4, the 42 unique adaptations presented in Table 2 were consolidated into 17 succinct adaptations representing 12 distinct groupings in the A‐FRAME matrix within the three thematic groups, described below.

#### 3.2.1 Project discontinuation

The thematic grouping project discontinuation was used to identify a project that was discontinued and resources reallocated for a different purpose. Two QI projects (C and D) were deprioritized in favor of COVID‐19‐related projects. In both instances, the only modification was the decision to stop the project, bringing both the intervention and evaluation to an end. Since the projects were not completed, the ability to achieve the intended goals and outcomes of the project were not realized.

#### 3.2.2 Intervention adaptation

Changes related to the intervention being implemented were classified under the intervention adaptation thematic group, which comprised of three subgroups: delivery, training, and staffing. Delivery includes the FRAME categories of what was being delivered (*content*), how it was being delivered (*context*), and strategies used to improve delivery (*implementation activities*). The FRAME's *training and evaluation* category was separated, with training regarded as a strategy to improve intervention uptake or a component of interventions, thus, a better fit with ‘intervention’ as a concept than evaluation.

Adaptations that restricted the hiring and training of care providers impeded the upskilling and capacity of the team to deliver the intervention, thus, were determined to impact implementation and the ability to deliver the intervention successfully. For example, Project G used a learning collaborative to teach dialysis providers to identify seriously ill patients and have quality goals of care discussions with them, but the pandemic precluded participants from traveling for the final learning session, so training was adapted to a virtual format, resulting in a reduction in attendance. In addition, the session attended to the effects of COVID‐19 on participating centers rather than the originally planned content, omitting training content to support goals of care discussions, a core function of the intervention.

Shifts to telehealth were considered adaptations to intervention delivery, which had mixed impacts on projects. For Project A, since the purpose of the intervention was to provide in‐person home care, the adjustment to telehealth altered the core function and goal by stifling the amount of care provided. Furthermore, new patients could not be enrolled, further limiting care access by homebound older adults. However, Project E's shift to telehealth, although challenging, allowed the provision of palliative care, the core function and goal of the intervention, to continue.

Additional intervention adaptations that did not alter core function were changes made to overall intervention delivery timelines or new safety protocols. For example, Project F's intervention was to provide palliative care to upper gastrointestinal cancer patients scheduled to undergo surgery, but because surgeries were postponed, the corresponding intervention was also paused. The intervention itself and the way it was delivered remained unchanged.

#### 3.2.3 Evaluation adaptation

As noted above, the FRAME category *training and evaluation* was divided into two categories, with evaluation adaptation becoming a stand‐alone thematic group as evaluation was perceived to be separate from the intervention. Evaluation adaptations included changes to study recruitment, data collection, and study design.

All evaluations were mixed methods, but the one project where the quantitative approach was modified was the only evaluation modification regarded as altering the core function. Project B's evaluation changed from a design to assess effectiveness with a comparison group to an observational study, which was perceived to be a critical change to what stakeholders would be able to conclude from the evaluation. Whereas the quantitative components of all other active projects remained unchanged, and the adjustments to the qualitative approaches were deemed not to alter the core function. In fact, the adaptations to the qualitative methods allowed for data to continue being collected not only to assess the implementation and effectiveness of interventions, but also document the effects of the pandemic that would otherwise be missing if only gathering quantitative data.

Commonly reported evaluation adaptations included changing the mode of data collection to remote methods (eg, telephone interviews) and pausing recruitment efforts, which typically corresponded to pauses in intervention delivery. Such changes were deemed not to impact the evaluation's goals because data were intended to be triangulated and used with other data sources, and evaluations were able to continue or continued after a delay. Some projects (A, E, G) added questions to their data collection tools (ie, interview guides, surveys) to explore the impact of the COVID‐19 pandemic on their study population. This was perceived to add value by exploring the context of implementation during the pandemic, thus were seen to enhance the explanatory value of evaluations.

## 4 DISCUSSION

The FRAME is comprehensive in capturing the who, what, when, how, and why of project adaptations, but how to organize that information to understand the impact of changes on projects is missing.^6^ We described an analysis process used across seven health services QI and research projects to make meaning out of systematically captured data on adaptations; we called the resulting analytic framework the “A‐FRAME.” This process identified that not all FRAME constructs and categories are necessary for assessing the impact of adaptations on projects and some adaptations within projects could be combined. When Haley and colleagues^16^ utilized the FRAME to describe adaptations of implementation strategies, the authors similarly included and excluded FRAME constructs. We applied a similar strategy and demonstrated that the inclusion and exclusion of selected FRAME constructs during the analysis process worked across seven different projects. Additionally, we incorporated a description of the original program or evaluation to better inform how the plans were impacted, which Miller and colleagues'^10^ also included in their framework for recording adaptations and modifications for implementation strategies. Conceptually consolidating the constructs into the *what*, *how*, *why*, and *impact* on core function, along with *project summary* that constitute the A‐FRAME matrix domains, as described and shown in Tables 3 and 4, cognitively reduced the burden of information on adaptations making it easier to explore and assess the impact of those adaptations on each project.

Comparing the impact of COVID‐19 on the evaluation plans of seven different projects was illuminating in terms of the value of mixed methods for evaluations. In particular, the inclusion of qualitative approaches appeared to add value to projects as most were able to pivot their data collection strategy to incorporate data to explore the impact of the pandemic on the intervention and understand changes in the organizational context of implementation. Qualitative research methods allow for responsive and flexible data collection and helps present a more complete and nuanced understanding of program outcomes and implementation.^17^, ^18^ We found that quantitative designs were less flexible and less able to capture the dynamic nature of the pandemic and its influence on implementation and outcomes. Qualitative evaluation methods in our sample were more amenable to adapting to the unexpected events that arose during the pandemic. Qualitative designs may therefore increase learning opportunities in research or QI,^19^ and enable project viability when unexpected hurdles arise.^18^

In our sample, two of the four QI projects were brought to an early end by the COVID‐19 pandemic. This is perhaps unsurprising as health care systems quickly shifted focus and resources to prepare for surges and rapid implementation of new policies and procedures.^20^, ^21^ QI projects are meant to be continuous, iterative, and flexible,^22^, ^23^ so when the pandemic struck, projects that did not directly address the most urgent health system concerns at the time had their funding and resources reallocated to more immediate needs. While this may seem negative, this likely indicates the flexibility of QI to adapt to the needs of the health system. This is in contrast to the research initiatives, which, though they did ultimately continue as planned, experienced delays due to health systems focusing on managing the COVID‐19 pandemic.

### 4.1 Limitations

We recognize that evaluation adaptations made up a large proportion of the adaptations captured. While we do not know if this is more than other evaluations ongoing at the same time experienced, it is likely a result of having researchers who are attuned to evaluation processes document evaluation and intervention changes. We attempted to capture adaptations in real time, rather than through retrospective interviews as might more often be the case with studies on adaptations (eg, 8, 24). This process may have identified more adaptations than if we had waited until the end of the study period, though our third analysis step of consolidating linked adaptations aimed to balance the salience of events due to recency with the importance of changes. We also experienced variability in opportunities to identify adaptations related to the cadence of communication between researchers and implementation teams. Some project researchers worked more closely with implementers, such as the partnered QI projects where researchers and implementers met weekly or more often. For other projects, such as one of the national studies, contact between researchers and implementers was less frequent. We therefore perceive that there may be variation between projects in the depth of knowledge about intervention changes due to the opportunistic observational approach used. The identification of adaptations was determined by individual researchers, and although the definition and adaptation categorization were reviewed and discussed, the nominated researcher on each project team made the final decision of whether an event was an adaptation or not. Ideally, intervention implementers would assist with tracking and/or reviewing adaptations. Adaptations were only systematically tracked during the first wave of the pandemic, and subsequent adaptations would likely provide further insight into project outcomes. Although the projects are from one academic research institution, the projects reflect local, regional, and national experiences of trying to implement different programs during the COVID‐19 pandemic. While the specific adaptations may not be generalizable, the process developed to capture and make sense of adaptations could be utilized by researchers in other settings.

## 5 CONCLUSION

The A‐FRAME analytic approach proposed in this paper may provide a useful framework for those conducting QI, evaluation, or research projects who wish to assess how adaptations in their projects may be interpreted in understanding project progress and outcomes. This analytic process enabled us to determine the individual impact of adaptations and consolidate related modifications to arrive at information which is easily digested and usable by implementers and researchers alike. Adaptations that impact the outcomes of projects are an inevitable part of implementation and evaluation processes, and the A‐FRAME offers a practical approach for making sense of those changes.

## CONFLICT OF INTEREST STATEMENT

The authors have no competing interests to declare.

## Supporting information


**Table S1:** Excerpts from FRAME project matrices.
